# Dr. Kinglake, in Explanation

**Published:** 1800-07

**Authors:** R. Kinglake

**Affiliations:** Chilton, super Peldon


### *To the Editors of the Medical and Phyfical Journal.*

Gentlemen,

THROUGH accidental delay, your Journal for April has but juft reached me; in that Number is a paper from Dr. Sherwen, in which he complains, in an epithet not lefs *harfh* than *unmerited*, of my having unneceffarily reflected on the correctnefs of his experience in the medicinal efficacy of Fox-glove.* Your indulging me with a few words in explanation, will probably adjuft the mifunderftanding, by convincing even the Doctor himfelf, that the remark alluded to, was not (to borrow his own language) "*unneceffary, and therefore not impertinent.*"

I repeat that the Doctor has, apparently, with the moft humane intention, cautioned againft the free ufe of Fox-glove; but that the ground of his dread, refpecting its producing finifter effects, exifts in his having not employed it fo extenfively as has occured to me to do in numerous inftances, without having witneffed any pernicious confequence to acrue from it.

With whatever benevolence of defign a medicine may be objected to, as being "*infidious*" in its operation, and *poffeffing* "*deleterious* and *poifonous properties*," the caution cannot be admitted as founded on *accurate experience*, when it is well known, that it may be adminiftered in all ages and temperaments, and in much larger dofes than many articles of the materia medica, *e. g.* corrofive muriate of quickfilver, arfenic, tartarifed antimony, &c. without inconvenience.

The moft falutary medicines, by abufe, may indifputably prove the moft deadly poifons; but, for that reafon, to decry them as poffeffing poifonous properties, and unfit for popular employ, might ferve injurioufly to check a due inveftigation of their powers, by thofe even who might be fully competent to the refearch.

The fubject of difpute then centers merely in, whether it be neceffary to denounce a medicinal fubftance of acknowledged falutary powers as poifonous, becaufe it might prove fo by injudicious ufe? or to rank it among the moft efficient agents, capable of being employed within certain limits, without any rifk of producing deleterious effects? In the latter eftimation I am inclined to hold Fox-glove, as moft conducive to the ultimate

* Vide Medical and Phyfical Journal, Vol. III. p. 122.

timate eſtabliſhment of its real powers. Nor, in my apprehenſion, is much to be feared from the popular uſe of this remedy, under the reſtrictions uſually promulgated relative to a powerful medicine. I can feel no alarm for a dropſical or conſumptive patient infuſing the leaves of Fox-glove in the form of tea, and beginning with a table ſpoon full of it, gradually increaſing the doſe to a common tea-cup full. The nauſea induced by a ſlight exceſs of this medicine, will operate as a ſafeguard to its undue adminiſtration. It requires, indeed, no ſmall addreſs to prevail on a patient who has been ſeverely ſickened by an over-doſe of Fox-glove, to recur to its uſe; hence it may be preſumed, that fewer inſtances of its proving poiſonous in domeſtic employ would occur, than of its arreſting and curing deſtructive diſeaſes.

Dr. Sherwen's avowed experience of the ſucceſsful uſe of the ſeeds of Fox-glove, is implicitly credited by me; the trial was judicious, and, to my knowledge at leaſt, original. It deſerves to be repeated, on the Doctor's ſtatement of their ſuperior efficacy. No opportunity ſhall eſcape me of ſubjecting them to the teſt of experiment; yet, a poſteriori, it muſt be confeſſed, that much identity between the ſeminal ſtructure and the organic development of vegetable ſubſtances, cannot be reaſonably expected. The various ſecretions, depoſitions, and arrangements which obtain in the fully evolved vegetable, do not preſent in the germen; a ſuſceptibility only for theſe effects, under ſuitable agency, exiſts in that embryon ſtate.

The medicinal efficacy of plants is probably referable to the conſtitution of principles, reſulting from the peculiar organic actions of the different parts of the vegetable fabric.

This remark is not intended to diſparage the authority of Dr. Sherwen's experience; it would appear from his teſtimony, that the powers of the Fox-glove were really concentrated in the ſeed. This fact would likewiſe warrant me in making trial of the roots of Fox-glove, as probably poſſeſſing alſo the native energies of the plant in ſuperior perfection.

I cannot approve of Dr. Sherwen's propoſal of drying the Fox-glove in a ſtrong expoſition to the ſun. Are not the ſenſible properties of all plants leaſt diminiſhed in drying, when the proceſs of exſiccation has been carried on in ſecluſion from ſolar light? And, on parity of reaſon, are not the remote and proximate arrangement of principles (on which medicinal efficacy muſt eſſentially depend) alſo liable to be more deranged by the decompoſing power of light, than by ſlow exhalation produced by moderate temperature in a dark ſituation?

It appears to me that a preferable mode for drying Fox-glove, in common with all other plants intended for medicinal uſe ufe, is, to ftrew the recent leaves on the furface of tin plates, and introduce them into an *oven* previoufly heated, and kept to about the temperature of eighty degrees of Fahrenheit's thermometer. If the mouth of the oven be ftopped during the procefs, every adventitious influence from light and air will be fufficiently precluded. In this heat the humidity of the plant will be fpeedily, equally, and uninterruptedly detached, leaving the elementary and conftituent principles in as near a ftate of native arrangement, as the ceffation of organic life can admit.

A cafe of hæmoptyfis, of three weeks ftanding, in a married lady, aged about twenty-five, under circumftances of ftrong conftitutional tendency to pulmonary confumption, has been lately cured by the tincture of Fox-glove under my direction; it afforded fpeedy relief; was taken during three weeks; more than thirty drops could not be borne without naufea. The pulfe occafionally intermitted under its influence; was raifed from a fmall, hard vibration, to much comparative foftnefs and fullnefs, but uniformly exceeded in frequency the natural ftandard.

It has alfo been fuccefsfully employed by me, within thefe few weeks, in two other inftances. One, an elderly man, fuffering under habitual afthma, with urgent cough and rapid emaciation. Not having any of the tincture at hand, the powder was fubftituted, in dofes of one grain three times a day. Its efficacy was ftrongly felt in the courfe of a week, in the abatement of cough, return of appetite, and other convalefcent changes. No naufea nor fenfible impreffion on the pulfe was produced, it fteadily retained a morbid hardnefs and frequency. The patient is now perfectly free from cough; refpires with tolerable freedom, but complains of undiminifhed debility.

The other was a boy, about fixteen years of age, who had been afflicted nearly two months with febrile paroxyfms, recurring every evening, cough, dyfpnœa, mucous expectoration, preternatural tenfion of the abdomen, and an icteric difcolouration of the whole fkin. The tincture was taken upwards of three weeks; not more than twenty drops could be endured; its operation was moft ftrongly foporific; the patient was rendered fo extremely and diftinctly comatofe by its agency, that, in a few minutes after taking it, he would irrefiftibly fall afleep, and that fo foundly, and durably, as to require at times to be actually roufed from his lethargic ftate. His difpofition to flumber, after the firft week of employing the Fox-glove, was fo infuperably ftrong, that he could not, without much effort, either raife his chin from his cheft, or open his eyes, to relate how he felt. He defcribed his fenfations, however, as being agreeable, and expreſſed his firm perſuaſion that he gained ground daily. The recurrence of the febrile paroxyſms was ſoon obviated, and every other ſymptom progreſſively amended, excepting the ſallow hue of the ſkin. The pulſe did not intelligibly correſpond with the effects produced, it was neither ſenſibly retarded, nor ſtrengthened, but continued morbidly quick and feeble. The friends of the patient deſiſted from proſecuting the uſe of the medicine, from a belief that his recovery was ſufficiently advanced.

An additional proof of the *inſufficiency* of Fox-glove, in the cure of the *laſt ſtage* of pulmonary conſumption, has been recently afforded by the fatal termination of the caſe of a young man under my care, to whom it had been freely exhibited in every variety of form, and under the moſt approved collateral regulations, during three months, with no farther benefit than temporary alleviation; latterly, it became quite inert, unleſs given in doſes of ſixty drops, when noxious ſickneſs occaſionally enſued. It was at length relinquiſhed as *uſeleſs*, when the ſubſtitution of ſmall doſes of opium, in a diluted ſolution of ſulphuric acid, taken at ſhort intervals, produced the moſt gratifying palliative effects. I am,

Gentlemen,

Your's, very reſpectfully,

*Chilton, ſuper Peldon,*

*June 19, 1800.*

R. KINGLAKE, M. D.

CRITICAL

